# Long‐Term Disease‐Free Survival After Systemic‐First Sequential Multimodal Therapy for Advanced and Recurrent Large‐Cell Neuroendocrine Carcinoma of the Uterine Cervix: A Case Report

**DOI:** 10.1111/jog.70341

**Published:** 2026-05-18

**Authors:** Kimio Wakana, Mayu Akita, Fumiko Tsubata, Yusuke Kori, Tamami Odai, Naoyuki Miyasaka

**Affiliations:** ^1^ Department of Obstetrics and Gynecology Institute of Science Tokyo Tokyo Japan; ^2^ Department of Perinatal and Maternal Medicine (Ibaraki) Institute of Science Tokyo Tokyo Japan; ^3^ Department of Women's Health Institute of Science Tokyo Tokyo Japan

**Keywords:** cervical cancer, chemoradiotherapy, large cell, lymph node metastases, neuroendocrine carcinoma

## Abstract

Cervical large‐cell neuroendocrine carcinoma (LCNEC) is a rare and highly aggressive malignancy with poor prognoses, particularly in advanced or recurrent disease. We describe a case of advanced and recurrent cervical LCNEC in which long‐term disease‐free survival was achieved following a systemic‐first, sequential multimodal treatment strategy. A 41‐year‐old woman presented with abnormal vaginal bleeding and was diagnosed with cervical LCNEC with pelvic and para‐aortic lymph node metastases. Neoadjuvant irinotecan–cisplatin chemotherapy resulted in marked tumor regression, enabling radical hysterectomy. Postoperative therapy consisted of etoposide–cisplatin chemotherapy, followed by pelvic and para‐aortic radiotherapy. Mediastinal lymph node recurrence developed 3 years later and was successfully treated using the same chemotherapy regimen combined with site‐directed radiotherapy, resulting in complete remission. The patient remained disease‐free 9 years after the final treatment completion. This case suggests that carefully sequenced systemic chemotherapy, surgery, and radiotherapy integration may provide durable disease control in selected patients with advanced or recurrent cervical LCNEC.

## 1 Introduction

Cervical large‐cell neuroendocrine carcinoma (LCNEC), classified as a neuroendocrine tumor in the 2020 World Health Organization classification, accounts for approximately 0.3% of cervical malignancies and is associated with aggressive clinical behavior and poor prognosis [1].

Although multimodal treatment combining surgery, chemotherapy, and radiotherapy is generally recommended, no standard treatment strategy has been established due to the limited number of reported cases. In particular, the optimal sequences and indications for neoadjuvant chemotherapy, surgery, and radiotherapy remain unclear.

Herein, we present a case of advanced and recurrent cervical LCNEC that achieved long‐term disease‐free survival after a sequential multidisciplinary treatment strategy integrating systemic chemotherapy, surgery, and radiotherapy.

## 2 Case Presentation

The patient was a 41‐year‐old woman (gravida 1, para 1) with a history of uterine fibroids and endometriosis, which were diagnosed at 20 years of age, without undergoing treatment. Menarche occurred at 15 years, and her menstrual cycles were regular, at approximately 30 days per cycle. She reported menorrhagia and dysmenorrhea, which were treated with nonsteroidal anti‐inflammatory drugs, and presented to our hospital with irregular vaginal bleeding.

Pelvic examination revealed a friable cervical tumor approximately 6 cm in diameter. No vaginal invasion was observed, and the uterus was enlarged to the size of her fist because of adenomyosis (Figure 1A). Digital rectal examination revealed no involvement of the cardinal ligaments. Histopathological examination of a cervical biopsy specimen confirmed LCNEC.

**FIGURE 1:**
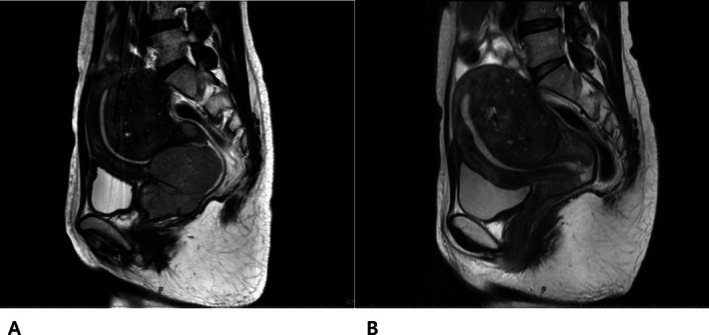
Pelvic magnetic resonance imaging findings before and after neoadjuvant chemotherapy (T2‐weighted sagittal images). (A) Before neoadjuvant chemotherapy, a cervical tumor measuring approximately 6 cm in diameter was observed. (B) After two cycles of neoadjuvant chemotherapy, the tumor decreased in size to approximately 35 mm in diameter.

18F‐FDG positron emission tomography‐computed tomography (FDG PET‐CT) demonstrated intense uptake in the primary cervical tumor (SUVmax 7.9) and mild uptake in the para‐aortic lymph nodes (SUVmax 2.5), consistent with nodal metastases, without evidence of distant organ metastases (Figure 2A). The clinical stage was IIIC2, according to the 2018 FIGO classification (T1b3N2M0, UICC 2021).

**FIGURE 2:**
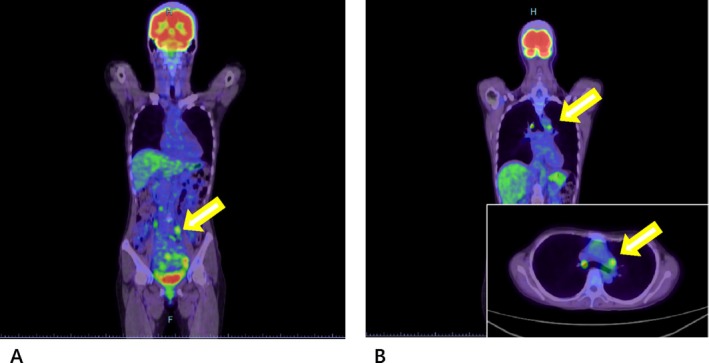
Positron emission tomography‐computed tomography findings at diagnosis and at recurrence. (A) Before neoadjuvant chemotherapy, para‐aortic lymph node uptake at diagnosis (arrows). (B) At recurrence, mediastinal lymph node uptake at recurrence (arrows).

Neoadjuvant irinotecan hydrochloride (CPT‐11) (60 mg/m^2^ on Days 1, 8, and 15) plus cisplatin (CDDP) (60 mg/m^2^ on Day 1) chemotherapy was administered for two cycles, reducing the tumor diameter to 35 mm (Figure 1B). Radical abdominal hysterectomy with bilateral salpingo‐oophorectomy and a para‐aortic lymph node biopsy were performed. Histopathological examination of the surgical specimen confirmed LCNEC of the cervix, with positive immunohistochemical staining for synaptophysin, chromogranin A, and CD56. In addition, diffuse p16 expression was observed, supporting an association with high‐risk human papillomavirus infection. These findings were consistent with the preoperative biopsy findings (Figure 3).

**FIGURE 3:**
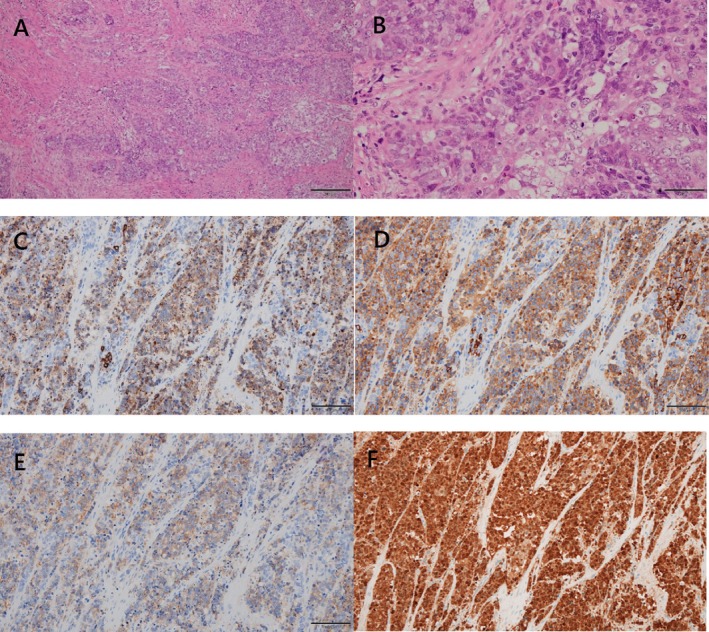
Histological findings of the surgical specimen of the cervical tumor. (A) H&E stain, original magnification × 10. (B) H&E stain, original magnification × 40, (C) Immunohistochemistry (original magnification × 20): Large‐cell neuroendocrine carcinoma (LCNEC) tumor cells are positive for chromogranin A. (D) Immunohistochemistry (original magnification × 20): LCNEC tumor cells are positive for synaptophysin. (E) Immunohistochemistry (original magnification × 20): LCNEC tumor cells are positive for CD56. (F) Immunohistochemistry (original magnification × 20): LCNEC tumor cells are positive for p16.

Postoperative chemotherapy with etoposide (100 mg/m^2^ on days 1–5) and cisplatin (75 mg/m^2^ on Day 1) was initiated for systemic disease control. From the second cycle, whole pelvic (50.4 Gy/28 fractions) and para‐aortic lymph node (39.6 Gy/22 fractions) irradiation were administered concurrently. To minimize treatment‐related toxicity during combined chemoradiotherapy, the chemotherapy doses were reduced to etoposide (100 mg/m^2^ on Days 1, 3, and 5) and cisplatin (60 mg/m^2^ on Day 1). A total of five cycles of chemotherapy were administered during the radiotherapy course. During the postoperative chemoradiotherapy, grade 4 neutropenia occurred without febrile episodes. Nevertheless, no treatment delay or discontinuation was required, and the planned treatment course was completed as scheduled.

FDG PET‐CT revealed mediastinal lymph node recurrence (SUVmax 3.5) 3 years and 1 month after the initial treatment (Figure 2B). Salvage CPT‐11/CDDP chemotherapy was initiated with the primary aim of systemic disease control, which is consistent with the postoperative treatment strategy. Radiotherapy to the mediastinum (60 Gy in 30 fractions) was subsequently incorporated in the second cycle to optimize locoregional control. Owing to prolonged myelosuppression, the chemotherapy dose was reduced to 90% from the third cycle onward. Despite the dose modification, the treatment was completed as planned with acceptable tolerability, and four cycles were administered during radiotherapy, resulting in a complete response.

The patient has remained disease‐free for 9 years and 2 months after the completion of the final treatment.

## 3 Discussion

Cervical LCNEC is a rare and highly aggressive malignancy characterized by early lymphovascular invasion and frequent nodal and distant metastases, resulting in poor prognoses, particularly in advanced or recurrent disease [2, 3, 4]. Owing to its rarity, optimal treatment strategies have not been established, and management is often extrapolated from small‐cell neuroendocrine carcinoma or pulmonary neuroendocrine tumors [2, 3, 4]. The present case is notable for the long‐term disease‐free survival despite advanced disease and recurrence.

Cervical LCNEC is considered a systemic disease, even in its early stages, emphasizing the importance of systemic chemotherapy [4]. Platinum‐based regimens, including etoposide or irinotecan, are commonly used, although no standard regimen exists [2, 3, 4, 5, 6]. Irinotecan–cisplatin was selected as neoadjuvant chemotherapy because this regimen has been reported to induce marked tumor regression in cervical LCNEC and may facilitate definitive surgical resection in selected patients [5].

Surgery after tumor reduction may improve cytoreduction and local control when complete resection is feasible [2, 3, 4, 5, 7]. In our patient, neoadjuvant chemotherapy followed by radical hysterectomy achieved adequate local control.

An important feature of the present case was the planned sequential integration of chemotherapy, surgery, and radiotherapy. In small‐cell neuroendocrine carcinoma of the cervix, etoposide‐cisplatin‐based chemoradiotherapy has been reported to achieve favorable outcomes, with a 3‐year overall survival rate of approximately 60% and a disease‐free survival rate approaching 80% in early‐stage disease, as demonstrated by Hoskins et al. [7] Therefore, we applied a strategy similar to that used for small‐cell neuroendocrine carcinoma.

In addition, the selection and sequencing of chemotherapy regimens in this case were based on a systemic‐first sequential multimodal strategy. Given the aggressive biological behavior of LCNEC and its propensity for early dissemination, systemic chemotherapy was prioritized to address potential micrometastatic disease, followed by local treatment for consolidation. In the postoperative setting, in which no macroscopic residual tumor was present, we adopted a planned sequential multimodal approach. Instead of standard concurrent chemoradiotherapy, systemic chemotherapy was administered first to prioritize systemic disease control, followed by radiotherapy to consolidate locoregional control. This systemic‐first sequential multimodal strategy was intended to target potential micrometastatic disease before initiating radiotherapy, thereby sequentially integrating systemic and locoregional treatment modalities in a tolerable manner [2, 3, 4, 5, 6, 7].

At recurrence, irinotecan‐cisplatin was reused because the tumor had shown a favorable initial response to this regimen, suggesting preserved chemosensitivity; in addition, systemic therapy remains central in recurrent neuroendocrine cervical carcinoma. Site‐directed radiotherapy was added for local consolidation. The same approach was effective for the mediastinal lymph node recurrence, and a complete response was achieved. Therefore, carefully sequenced multimodal therapy may provide durable disease control in selected patients.

Management of recurrent LCNEC remains challenging [2, 3, 4, 7]. In the present case, the isolated recurrence responded to combined chemotherapy and radiotherapy, supporting aggressive salvage therapy for selected cases of oligometastatic disease.

Compared with previously reported cases, the present case was characterized by a consistently applied systemic‐first treatment sequence. While surgery‐first management or etoposide‐cisplatin‐based chemoradiotherapy has commonly been reported in cervical neuroendocrine carcinoma [1, 2, 3, 4, 5, 6, 7, 8], our patient received irinotecan–cisplatin as neoadjuvant therapy, etoposide‐cisplatin as postoperative systemic therapy followed by radiotherapy, and irinotecan–cisplatin was reused at recurrence on the basis of the favorable initial response. Such a deliberately planned and consistently applied sequential multimodal strategy, maintained across both the primary and recurrent settings, has rarely been described in the literature.

Previous reports have demonstrated a median overall survival of approximately 16 months for cervical LCNEC, and neuroendocrine carcinoma of the cervix overall has a mean recurrence‐free survival of only around 16 months [3, 4]. In addition, survival outcomes for stage III disease remain poor, and durable remission after recurrence is rarely achieved.

Although rare, some cases of relatively prolonged survival have been reported. For instance, Tangjitgamol et al. described a patient with cervical LCNEC treated with paclitaxel and carboplatin who survived for 44 months [9].

In this context, long‐term disease‐free survival exceeding 9 years in patients with stage IIIC2 cervical LCNEC is exceedingly rare, given the historically poor prognosis associated with advanced‐stage neuroendocrine carcinoma. The reported survival rates for stage III disease remain limited, and durable remission after recurrence is uncommon. In this context, the present case represents an exceptional clinical course.

Although limited to a single case, sequential multimodal treatment integrating systemic chemotherapy, surgery, and site‐directed radiotherapy may represent a potential treatment option for achieving durable disease control in patients with advanced or recurrent LCNEC. An accumulation of cases and multicenter studies are needed to establish optimal management strategies.

## Author Contributions

**Kimio Wakana:** conceptualization, writing – original draft, writing – review and editing, investigation. **Mayu Akita:** validation. **Fumiko Tsubata:** validation. **Yusuke Kori:** validation. **Tamami Odai:** validation. **Naoyuki Miyasaka:** validation, supervision.

## Disclosure

Part of this work was presented at the Annual Meeting of the Japanese Society of Gynecologic Oncology, which was held in Sendai, Japan, on January 29–30, 2021.

## Ethics Statement

This study was approved by the Institutional Review Board of our hospital (approval number: M2015‐524).

## Consent

Informed consent was received from the patient.

## Conflicts of Interest

The authors declare no conflicts of interest.

## Data Availability

The data supporting the findings of this study are not publicly available due to ethical and privacy considerations, as they contain potentially identifiable patient information. As this is a rare case, there is an increased risk of indirect patient identification even after anonymization. Therefore, the data are not deposited in a public repository. Limited data may be available from the corresponding author upon reasonable request and with approval from the institutional ethics committee.
